# Herbal medicine *Banha-sasim-tang* for the treatment of functional dyspepsia protocol for a systematic review of randomized controlled trials

**DOI:** 10.1097/MD.0000000000015760

**Published:** 2019-05-31

**Authors:** Seok-Jae Ko, Soo-ho Cho, Keum-Ji Kim, Jin-sung Kim, Na-yeon Ha, Jae-Woo Park

**Affiliations:** Department of Gastroenterology, College of Korean Medicine, Kyung Hee University, Seoul, Republic of Korea.

**Keywords:** *Banha-sasim-tang*, functional dyspepsia, meta-analysis, protocol, systematic review

## Abstract

**Background:**

Functional dyspepsia (FD) has a high prevalence rate. The dyspeptic symptoms are not easily cured despite the availability of various conventional Western medical treatments. *Banha-sasim-tang* (BST) is a traditional herbal medicine that has long been used for treating FD.

**Methods:**

The following databases will be searched from inception to January 2019: Medline via PubMed, the Cochrane Central Register of Controlled Trials, EMBASE, Allied and Complementary Medicine Database, National Digital Science Library, Korean Medical Database (KoreaMed), Oriental Medicine Advanced Searching Integrated System, Korean Studies Information Service System, China National Knowledge Infrastructure Database, and Citation Information by Nii. Randomized controlled trials (RCTs) that used BST or herbs-added BST for treating FD will be included in the systematic review. Control groups in these RCTs will be the placebo, no-treatment, and conventional Western medicine groups. RCTs that compared BST and Western medicine combination therapy with the conventional Western medicine will also be included in the systematic review to investigate the synergistic effect of BST and Western medicine. Data extraction and evaluation of risk of bias will be performed by 2 independent investigators. The primary outcome will be the total clinical effective rate and secondary outcomes will include gastrointestinal symptom scale, visual analog scale, FD-related quality of life, electrogastrography, plasma motilin, dyspepsia-related symptom score, gastric emptying, and adverse events. RevMan version 5.3 will be used for data integration and analysis.

**Results:**

This systematic review will provide a high-quality integration of current evidence of BST for treating FD from several aspects including total clinical effective rate, dyspepsia-related symptoms, quality of life, and adverse events.

**Conclusions:**

This systematic review will provide evidence of the effectiveness and safety of BST on FD.

**Ethics and dissemination:**

Identifying information of the participants will not be revealed; hence, this protocol does not need ethical approval. The systematic review will be published in a peer-reviewed journal and disseminated electronically.

**Trial registration number:**

PROSPERO CRD42019123285.

## 1 Introduction

Functional dyspepsia (FD) is a medical condition that poses a significant impact on the daily activities of patients and is characterized by 1 or more of dyspeptic symptoms, such as postprandial fullness, early satiation, epigastric pain, and epigastric burning, which remain unexplained even after routine clinical evaluation.^[1]^ Delayed gastric emptying or antral dysmotility is considered to be the main cause of these dyspepsia symptoms in FD patients.^[4]^ FD is not a life-threatening disorder and is not associated with increased mortality; however, the prevalence of FD is very high and greatly affects the patients’ quality of life. Research has shown that the true global prevalence of FD is estimated between 11.5% and 29.2%.^[3]^ Currently, FD can be treated by conventional medications including acid-suppressive drugs, prokinetic agents, and antidepressants. Despite of these treatments, dyspeptic symptoms are not easily cured in many patients.

*Banha-sasim-tang* (BST), which is also known as *Banxia-xiexin-tang* in traditional Chinese medicine and *Hange-shashin-to* in Kampo medicine, is an herbal medicine containing 7 herbs: *Pinelliae tuber*, *Scutellariae radix*, *Zingiberis rhizoma*, *Ginseng radix*, *Glycyrrhizae radix*, *Zizyphi fructus*, and *Coptidis rhizome*. It has been used in the Korean medicine for the treatment of epigastric stuffiness, which is similar to the symptoms of FD.^[2]^ According to the previous study, BST or *P tuber*, a major component in BST, may improve the impaired gastric motility in the FD patients by changing the levels of gut hormones and regulating the hypothalamic–pituitary–adrenal axis, thereby alleviating the early satiety symptoms.^[5]^

Several systematic reviews and meta-analyses describing the safety and efficacy of BST in the treatment of FD have been published.^[6–10]^ However, most studies compared BST with conventional Western medicines,^[7]^ and some reviews compared BST and herbal medicine combination, and not BST alone, with conventional Western medicine.^[8–10]^

As noted earlier, results from the previous reviews did not appear to provide adequate evidence to support the recommendations regarding the use of BST for the treatment of FD. The purpose of this review is to systematically synthesize the primary studies that investigate the efficacy and safety of BST on FD.

## 2 Methods and analysis

### 2.1 Inclusion criteria for study selection

#### 2.1.1 Types of studies

This systematic review will include randomized controlled trials (RCTs) and quasi-RCTs. Animal studies, case reports, and commentaries will be excluded.

#### 2.1.2 Types of patients

The patients diagnosed with FD on the basis of the ROME criteria will be included in this systematic review regardless of their age, gender, and race. The ROME criteria are the diagnostic criteria used for the functional gastrointestinal (GI) disorders. It was first announced in 1992, and in 2016, ROME IV criteria were finalized after some revisions. If studies were published before 1991, they will be judged based on the criteria similar to the ROME criteria (eg, uninvestigated dyspepsia). The judgment will be performed along with the consensus of the 2 reviewers (SK and SC). Secondary GI symptoms such as gastroesophageal reflux disease and irritable bowel syndrome will be excluded according to the ROME criteria.

#### 2.1.3 Types of interventions

Randomized studies involving BST and herbs-added BST will be included. Studies comparing BST with the following types of control interventions will be included: conventional Western medicines such as proton-pump inhibitors, prokinetics, GI tract regulators, and antispasmodics; placebo BST that has the same color and odor as BST; and no-treatment, waiting. Additionally, this review will also investigate the effect of BST–Western medicine combination therapy compared with Western medicine alone.

#### 2.1.4 Types of outcome measures

Primary outcome will include the total clinical effective rate. Secondary outcomes will include GI symptom scale, visual analog scale, FD-related quality of life, electrogastrography, plasma motilin, dyspepsia-related symptom score, gastric emptying, and adverse events.

### 2.2 Data sources

The following databases will be searched from inception to January 2019: Medline (via PubMed), Cochrane Central Register of Controlled Trials (CENTRAL), EMBASE, and Allied and Complementary Medicine Database (AMED). Five Korean medical databases will also be searched, which are as follows: National Digital Science Library (NDSL), Korean Medical Database (KMbase), KoreaMed, Oriental Medicine Advanced Searching Integrated System (OASIS), and Korean Studies Information Service System (KISS). We will also search other Asian databases such as China National Knowledge Infrastructure Database (CNKI) in Chinese and Citation Information by Nii (CiNii) in Japanese. Trial registries such as clinicaltrials.gov and Clinical Research Information Service will also be included.

The search terms will consist of disease and intervention-related terms. The disease-related terms will include the “intestine,” “digestion,” “stomach,” “gut,” “dyspepsia,” “discomfort,” “disturbance,” “pain,” and “dysfunction.” The intervention-related terms will be “*Banha-sasim-tang*,” “*Banxia-xiexin-tang*,” “*Hange-shashin-to*,” “herbal medicine,” “medicine,” and “botanical.” The search strategies designed for Medline (via PubMed) are presented in Table 1. Modified search strategies will be applied to the other databases. No language restriction will be imposed.

**Table 1:**
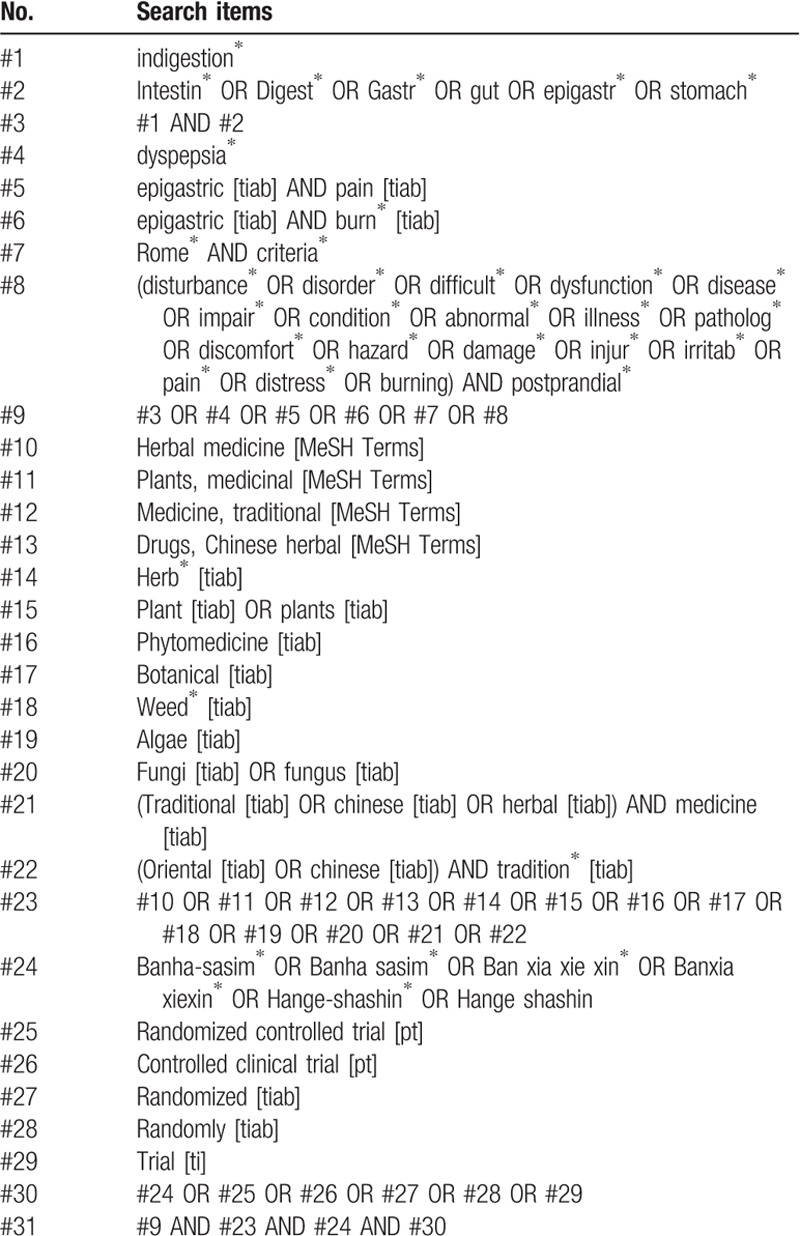
Search strategy used in PubMed.

### 2.3 Data collection and analysis

#### 2.3.1 Study selection

**Figure 1:**
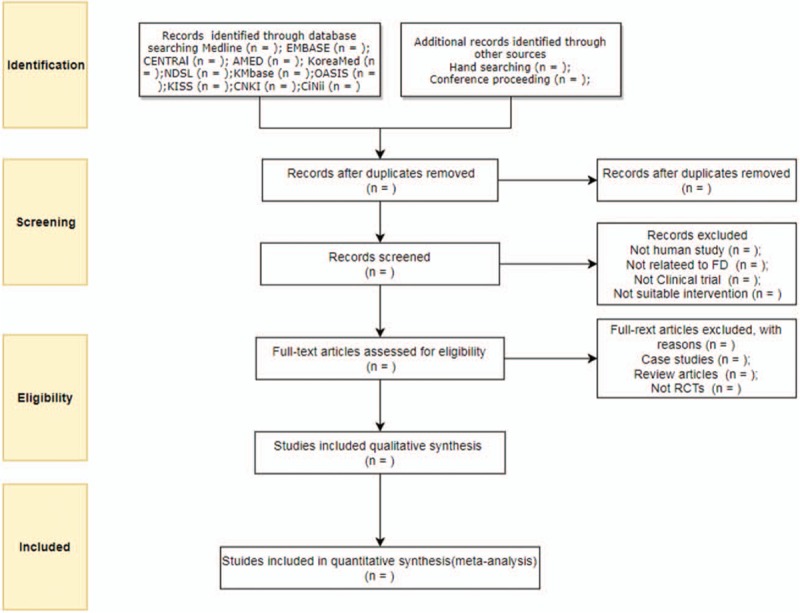
Flow chart of the search process.

Two reviewers (SK and SC) trained in the process and purpose of selection will independently review the title, abstract, and manuscript of all the studies to check for their eligibility to be included in the analyses. All studies searched by both electronic and hand methods will be uploaded to Endnote X8 (Clarivate Analytics, Philadelphia). The reason for excluding the study will be recorded and shown in PRISMA flow chart (Fig. 1). All disagreements will be resolved by a consensus and discussion between the 2 reviewers. If the agreement is not successful between the 2 reviewers, the arbiter (JK) will intervene and resolve the problem.

#### 2.3.2 Data extraction and management

Two review writers (KK and NH) will independently extract the data and fill the standard data extraction form, which will include the study information such as the first author, publication year, research design, interventions, treatment period, and outcomes. All differences between the 2 reviewers will be resolved by a consensus and discussion; however, if necessary, the arbiter (JK) will intervene to reach an agreement.

#### 2.3.3 Assessment of the risk of bias in the included studies

Two reviewers (KK and NH) will assess the risk of bias (RoB) based on the Cochrane collaboration's tool, which includes random sequence generation (selection bias), allocation concealment (selection bias), blinding of participants and personnel (performance bias), blinding of outcome assessment (detection bias), incomplete outcome data (attrition bias), selective reporting (reporting bias), and other bias. Assessment result will be shown through 1 of the 3 categories: low, unclear, and high. All disagreements will be resolved by a consensus and discussion between the 2 reviewers, and if necessary, the arbiter (JK) will intervene.

#### 2.3.4 Methods of measurements

Dichotomous data will be evaluated through the relative risk with 95% confidence intervals (CIs), and continuous data will be evaluated by the mean difference with 95% CIs.

#### 2.3.5 Unit of analysis issue

To avoid carry-over effects, we will use only the 1st-phase data in case of the randomized cross-over trials. If the trials have multiple intervention groups, a pair-wise comparison will be made.

#### 2.3.6 Dealing with missing data

In case of missing data or difficulty in finding enough data, we will try to contact the original investigators via e-mail to obtain sufficient data. Despite these attempts, if we are unable to obtain the data, it will be sought from the original source or trial reports. Statistical analysis will be performed based on the intent-to-treat principle.

#### 2.3.7 Heterogeneity assessment

Random effects model will be used for meta-analysis. We will use *I*^2^ statistics and *χ*^*2*^ test to assess the heterogeneity. *I*^2^ ≥ 50% and *P* value <.10 will indicate substantial heterogeneity.

#### 2.3.8 Assessment of publication bias

If the analysis includes more than 10 studies, a funnel plot will be generated to evaluate the publication bias or small-study effects.

#### 2.3.9 How to synthesize the data

We will use the review manager program (V5.3.5 Copenhagen: The Nordic Cochrane Centre, The Cochrane Collaboration, 2014) to perform the statistical analyses. All studies will be synthesized according to the type of intervention and/or control as follows: BST vs no treatment, BST vs placebo control, BST vs conventional Western medicine, and BST–Western medicine combined therapy vs conventional Western medicine alone. The herbs-added BST will be included in the BST group as described in the “Types of intervention” section.

#### 2.3.10 Subgroup analysis

In case of availability of enough subgroup studies to investigate the cause of heterogeneity, subgroup analysis will be performed. Its criteria will include pattern identification in Traditional Chinese Medicine, physical form of BST, number and type of added herbs, and treatment duration. If the quality of the study is judged to be low after the subgroup analysis, these studies would be removed to confirm the robustness of the results.

#### 2.3.11 Sensitivity analysis

We will use “the consolidated standards of reporting trials extension for herbal interventions” to evaluate the methodological and reporting quality of the studies, and the sensitivity analysis will be performed to evaluate the robustness of the results obtained from the meta-analysis.

#### 2.3.12 Grading the quality of evidence

We will use “The Grading of Recommendations Assessment, Development and Evaluation” to examine the quality of evidence.

## 3 Discussion

FD, a relapsing and remitting disorder, is the most common cause of dyspepsia.^[14]^ Up to 40% of patients with FD consult a physician,^[15]^ and FD has negative effects on an individual's work productivity.^[16]^ It also poses substantial financial implications for the patients. In the United States, the total medical costs associated with FD exceeded $18 billion in 2009.^[17]^

BST has been used in the traditional Korean medicine to treat GI diseases including FD.^[13]^ According to the recent research, BST regulates the GI function in the patients suffering from FD and also relieves the symptoms of GI cancer patients, such as nausea, vomiting, and anorexia.^[12,13]^ A study that investigated the pharmacokinetics of BST has shown that BST increases the somatostatin-immunoreactive substances and motilin-immunoreactive levels. Furthermore, the increase in the somatostatin-immunoreactive substances and motilin-immunoreactive levels contribute to the regulation of GI motility by accelerating gastric emptying.^[11]^

Several previous studies have investigated the effect and safety of BST on FD. One meta-analysis involving 9 studies has shown that *Ban Xia Xie Xin* decoction may have a better effect and may be safer for the patients suffering from FD as compared to the prokinetic agents. The incidence of adverse events such as GI symptoms and headache were observed in the control group; however, no side effects were observed in the *Ban Xia Xie Xin* decoction group.^[7]^ One systematic review involving 37 studies and having high heterogeneity showed that Chinese herbal medicine including *Ban Xia Xie Xin* decoction may have a better effect on FD than conventional Western medicine treatment, such as that using prokinetic agents, H_2_ receptor antagonists, and antidepressants, and may have no side effects.^[8]^ Another systematic review that involved 20 studies showed that Chinese herbal medicine, including *Ban Xia Xie Xin* decoction, may have a better effect on FD than the conventional Western medicine treatment, such as that using domperidone, trimebutine, mosapride, and omeprazole, and without side effects.^[9]^

Although these 2 systematic reviews involved other herbal medicines as well as BST, they demonstrated that BST can be effective for treating FD. As limitations of previous systematic reviews, most of included studies were Chinese and had poor methodological quality. Moreover, the variables were only focused on subjective parameter and the synergetic effect of herbal–Western medicine was not investigated. From this study, we will investigate the evidence that BST and herbs-added BST can be used for the treatment of FD through subjective and objective variables. In addition, efficacy and safety of BST will be investigated through RCTs that compared BST with no treatment, BST with placebo, BST with conventional Western medicine, and BST–Western medicine combined therapy with conventional Western medicine alone.

This review will provide an update on the recent RCTs involving BST and will be the first systematic review to focus on the efficacy and safety of BST, herbs-added BST, and BST with conventional Western medicine for the treatment of FD. We expect that this study will offer detailed information that is relevant to the clinical practice.

Any form of ethical approval is not required because the protocol for this review involves the collection of the published data. The systematic review will be published in a peer-reviewed journal and disseminated electronically or in print.

## Author contributions

**Conceptualization:** Seok-Jae Ko, Soo-ho Cho.

**Data curation:** Seok-Jae Ko, Soo-ho Cho, Keum-Ji Kim, Jin-sung Kim, Na-yeon Ha, Jae-Woo Park.

**Formal analysis:** Jin-sung Kim.

**Investigation:** Keum-Ji Kim, Jin-sung Kim, Na-yeon Ha.

**Methodology:** Keum-Ji Kim, Jin-sung Kim, Na-yeon Ha.

**Resources:** Jae-Woo Park.

**Writing – original draft:** Soo-ho Cho.

**Writing – review & editing:** Seok-Jae Ko.

Jae-Woo Park orcid: 0000-0002-1862-7317.
